# Correlation of High-Grade Osteosarcoma Response to Chemotherapy with Enhanced Tissue Immunological Response: Analysis of CD95R, IFN-γ, Catalase, Hsp70, and VEGF

**DOI:** 10.1007/s00428-024-03801-z

**Published:** 2024-05-15

**Authors:** Sjahjenny Mustokoweni, Ferdiansyah Mahyudin, Rosy Setiawati, Dian Nugrahenny, Mohamad Hidayat, Handono Kalim, Karyono Mintaroem, Loeki Enggar Fitri, Pancras C. W. Hogendoorn

**Affiliations:** 1 Doctoral Program in Medical Sciences, Faculty of Medicine, Universitas Brawijaya, Malang, Indonesia; 2 Department of Anatomical Pathology, Faculty of Medicine, Universitas Airlangga/Dr, Soetomo General Academic Hospital, Mayjen Prof. Dr. Moestopo 6-8, Airlangga, Gubeng, Surabaya, East Java Indonesia; 3 Department of Orthopaedic Surgery and Traumatology, Faculty of Medicine, Universitas Airlangga/Dr, Soetomo General Academic Hospital, Surabaya, Indonesia; 4 Department of Radiology, Faculty of Medicine, Universitas Airlangga/Dr, Soetomo General Academic Hospital, Surabaya, Indonesia; 5 Department of Pharmacology, Faculty of Medicine, Universitas Brawijaya, Malang, Indonesia; 6 Department of Orthopaedic Surgery and Traumatology, Faculty of Medicine, Universitas Brawijaya/Dr. Saiful Anwar General Hospital, Malang, Indonesia; 7 Department of Internal Medicine, Faculty of Medicine, Universitas Brawijaya/Dr. Saiful Anwar General Hospital, Malang, Indonesia; 8 Department of Anatomical Pathology, Faculty of Medicine, Universitas Brawijaya, Malang, Indonesia; 9 Department of Parasitology, Faculty of Medicine, Universitas Brawijaya, Malang, Indonesia; 10 Department of Pathology, Leiden University Medical Center, Leiden, The Netherlands

**Keywords:** Angiogenesis, Bone, Bone neoplasm, Immune response, Malignancy

## Abstract

High-grade osteosarcoma, a primary malignant bone tumour, is experiencing a global increase in reported incidence with varied prevalence. Despite advances in management, which include surgery and neoadjuvant chemotherapy often an unsatisfactory outcome is found due to poor or heterogeneous response to chemotherapy. Our study delved into chemotherapy responses in osteosarcoma patients and associated molecular expressions, focusing on CD95 receptor (CD95R), interferon (IFN)-γ, catalase, heat-shock protein (Hsp)70, and vascular endothelial growth factor (VEGF). Employing immunohistochemistry and Huvos grading of post-chemo specimens, we analysed formalin-fixed paraffin-embedded (FFPE) osteosarcoma tissue of resected post-chemotherapy specimens from Dr. Soetomo General Academic Hospital in Surabaya, Indonesia (DSGAH), spanning from 2016 to 2020. Results revealed varied responses (poor 40.38%, moderate 48.08%, good 11.54%) and distinct patterns in CD95R, IFN-γ, catalase, Hsp70, and VEGF expression. Significant differences among response groups were observed in CD95R and IFN-γ expression in tumour-infiltrating lymphocytes. The trend of diminishing CD95R expression from poor to good responses, accompanied by an increase in IFN-γ, implied a reduction in the count of viable osteosarcoma cells with the progression of Huvos grading. Catalase expression in osteosarcoma cells was consistently elevated in the poor response group, while Hsp70 expression was highest. VEGF expression in macrophages was significantly higher in the good response group. In conclusion, this study enhances our understanding of immune-chemotherapy interactions in osteosarcoma and identifies potential biomarkers for targeted interventions.

## Introduction

High-grade osteosarcoma is a primary malignant bone tumour characterized by the formation of immature bone or osteoid. It predominantly occurs in the long bone metaphysis, such as the distal femur, proximal tibia, fibula, proximal humerus, and pelvis, where the epiphyseal growth plate is highly active [1]. The reported incidence of osteosarcoma tends to increase globally, with varying prevalence in different regions [2]. In countries with less developed cancer registries, or public health care, the incidence remains unclear. For instance, a study at Dr. Cipto Mangunkusumo National Central General Hospital, Jakarta, (CMNCGH) reported an average of 16.8 cases per year with a total of 219 cases from 1995—2008 [3]. The National Health Survey in 2018 indicated a cancer prevalence of 1.79 per thousand people in Indonesia [4]. In contrast in the United States, the annual incidence ranges from 4 to 5 cases per million individuals [1].

The management of osteosarcoma involves surgery and chemotherapy. Chemotherapy protocols typically include combinations of cisplatin, doxorubicin, methotrexate, and ifosfamide [5]. Neoadjuvant chemotherapy, given before definitive surgery, often yields unsatisfactory results, with a significant number of patients exhibiting a poor histological response [6, 7].

At CMNCGH, among 20 stage IIB osteosarcoma cases, 12 cases (60%) showed unresponsiveness based on the Huvos grading system I and II during a time window from 1995 to 2008 [3, 8]. International data also reveals variations, with studies indicating substantial rates of unresponsiveness. In the United States, a study found a 31% response rate to induction chemotherapy (MAP regimen involving Methotrexate, Cisplatin, and Doxorubicin) and a 36% response rate with the addition of Ifosfamide (MAPI regimen) [9]. Meta-analyses in China and studies in Korea demonstrated varying response rates to intensified and conventional chemotherapy doses [10–12]. Similarly, studies in Tunisia indicated a high percentage (78%) of poor responses to chemotherapy indicated by tumour necrosis < 90% [13]. These findings raise concerns about the potential ability of cancer cells to protect themselves from chemotherapy agents. Osteosarcoma at first sight is not hallmarked by immune infiltrate, though when one observes more carefully, in the background of this tumour numerous macrophages can be found. This is also reflected in the expression signature in cDNA expression arrays [14]. Potentially this pathway can also be used to target the tumour as well as via an augmentation of the immune system for instance via the augmentation of natural killer cell activity [15]. Alternatively, the intact interferon signalling in leukocytes of osteosarcoma patients gives potential adjuvant treatment options [16].

Understanding the mechanisms behind the differences in the response of osteosarcoma to therapy is crucial for determining treatment success. Efforts to investigate these differences are necessary for the development of effective therapies and prognosis determination.

## Materials and Methods

In this study, we employed an observational approach with a retrospective design to assess the expression of CD95 receptor (CD95R), interferon (IFN)-γ, catalase, heat-shock protein (Hsp)70, and vascular endothelial growth factor (VEGF) in osteosarcoma patients. The evaluation involved a comparison based on the Huvos grading system (I, II, and III-IV) and immunohistochemical assessment methods. Our analysis utilized stored formalin-fixed paraffin-embedded (FFPE) osteosarcoma tissue from patients diagnosed with osteosarcoma with or without metastasis at DSGAH. Diagnoses were histopathologically confirmed at DSGAH during the 5 years of inclusion from 2016 to 2020.

The criteria for sample selection included lesion location in long bones, surgical procedures involving resection or amputation, receipt of neoadjuvant chemotherapy with Doxorubicin and Cisplatin only, sufficient tumour tissue in paraffin blocks for immunohistochemical examination, and the evaluation of chemotherapy response using the Huvos grading system. Exclusion criteria comprised non-representative samples in terms of the number of cells to be evaluated, biopsy as the sole type of surgery, patients undergoing life-saving amputation without preoperative chemotherapy, and patients receiving radiotherapy. The evaluation of Huvos grading [8] involved categorizing responses as per Garcia-Castelano’s classification [17]. Huvos grade I indicated a poor response with no necrosis or less than 50% necrosis, grade II reflected a moderate response with necrosis ranging from 50 to 90%, grade III signified a good response with necrosis between 90 and 99%, and Grade IV represented a total response with 100% necrosis.

We have evaluated FFPE tissue from 52 osteosarcoma patients treated by surgery both amputation and limb-sparing surgery. No biopsy specimens were used. For the assessment of IFN-γ expression in osteosarcoma cells, positive reactions were determined using anti-human IFN-γ (Cat. No. A12450, ABclonal, Woburn, MA, United States). Similarly, the examination of CD95R expression involved evaluating positive reactions in lymphocyte cytoplasm with the use of anti-CD95R (Cat. No. A19582, ABclonal, Woburn, MA, United States). Catalase activity in the tumour cell cytoplasm was analysed through the application of anti-Catalase (Cat. No. A11220, ABclonal, Woburn, MA, United States). The assessment of Hsp70 expression entailed the examination of positive reactions in the tumour cell cytoplasm using anti-Hsp70 (Cat. No. CM 407 A, Biocare Medical, Concord, CA, United States). Meanwhile, the analysis of VEGF expression involved evaluating positive reactions in macrophage cytoplasm using anti-VEGF (Cat. No. A17877, ABclonal, Woburn, MA, United States). Each section was treated with a labelled antibody, and diaminobenzidine served as the chromogen (Cat. No. BDB2004, Biocare Medical, Pacheco, CA, United States). After counterstaining, the sections underwent dehydration with increasing concentrations of alcohol and were mounted. All quantifications were conducted by evaluating five high-power field hotspots on lymphocytes through an Olympus CX41 light microscope with a magnification of 600 × and on tumour tissue through an Olympus CX41 light microscope with a magnification of 400x. The positive result indicated a continued activity of certain proteins associated with proliferation in malignant cells or mechanism of tumour resistance. Positive staining was only considered when cell bound; indeed there was sometimes some aspecific staining present in the extracellular matrix, but this was not considered positive or relevant.

The normality of the data was examined using the Shapiro–Wilk test. To compare the differences between groups, the Kruskal–Wallis test was employed, followed by Dunn's multiple comparisons tests. Additionally, Spearman's correlation test was executed. A *p*-value below 0.05 was deemed statistically significant. All statistical analyses were performed using GraphPad Prism for Windows, Version 9.3.0, San Diego, CA, United States.

## Results

The characteristics of the observed subjects are presented in Table 1. Chemotherapy responses in osteosarcoma patients were categorized as poor (Huvos grade I) at 40.38%, moderate (Huvos grade II) at 48.08%, and good (Huvos grade III) at 11.54%. The occurrence of osteosarcoma in the subjects of this study was found to be highest in the age range of 10–19 years and lowest in those above 30 years. The results of the Chi-square analysis for gender and age data revealed homogeneity, as evidenced by a *p*-value > 0.05. This suggests that gender and age did not serve as confounding factors within the scope of this study. Table 2 shows that in the poor response group, the highest number was the chondroblastic variant, with more females than males. This supports previous observations in studies that prove the chondroblastic type osteosarcoma has a poorer histological response to chemotherapy [18, 19]. This was followed by the osteoblastic variant, which was also more common in females. In the moderate response group, the osteoblastic and chondroblastic variants were balanced, with more males than females for both variants, followed by the fibroblastic variant, which was more prevalent in females. In the good response group, the osteoblastic and giant cell-rich variants were equally distributed in males, while in female patients, none of them showed a good response.

**Table 1:** Subject characteristics and chemotherapy response of osteosarcoma

Characteristics	Total (n = 52)	Huvos Grade 1 (n = 21)	Huvos Grade 2 (n = 25)	Huvos Grade 3 (n = 6)	*p-*value
Gender					
Male	30 (57.7%)	10 (19.2%)	15 (28.8%)	5 (9.8%)	0.458
Female	22 (42.3%)	11 (22.1%)	10 (19.2%)	1 (1.9%)	
Age (year)					
0–9	4 (7.7%)	2 (3.8%)	2 (3.8%)	0	0.469
10–19	38 (73.0%)	12 (23.0%)	20 (38.4%)	6 (11.5%)	
20–29	9 (17.3%)	6 (11.5%)	3 (5.7%)	0	
30–39	1 (2.0%)	1 (1.9%)	0	0	
40–49	0	0	0	0

**Table 2:** Distribution of chemotherapy response in various osteosarcoma subtypes based on gender

Chemotherapy Response	Osteosarcoma Subtypes	Male (n = 30)	Female (n = 22)	Total (n = 52)
Huvos Grade I	Osteoblastic	1	4	5
	Chondroblastic	5	6	11
	Fibroblastic	3	0	3
	Giant cell rich	1	1	2
	Telangiectatic	0	0	0
Huvos Grade II	Osteoblastic	7	3	10
	Chondroblastic	7	3	10
	Fibroblastic	1	4	5
	Giant cell rich	0	0	0
	Telangiectatic	0	0	0
Huvos Grade III	Osteoblastic	2	0	2
	Chondroblastic	1	0	1
	Fibroblastic	0	0	0
	Giant cell rich	2	0	2
	Telangiectatic	0	1	1

**Fig. 1:**
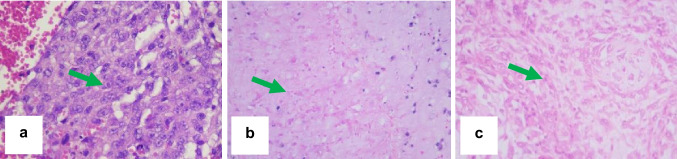
Histopathological examination based on Huvos grading. Huvos grade I shows viable tumour cells at 80% (a). Huvos grade II exhibits viable tumour cells at 40% with a necrotic area of 60% (b). Huvos grade III reveals viable tumour cells at 10% with a necrotic area of 90% (c). Haematoxylin–eosin stain, magnification 400x

Histological examination based on Huvos grading indicates that in Huvos grade I, viable tumour cells constitute 80%. Huvos grade II displays viable tumour cells at 40%, accompanied by a necrotic area of 60%. In Huvos grade III, viable tumour cells are observed at 10%, with a predominant necrotic area of 90% (Fig. 1).

Morphological changes that are pronounced in the pre-operative chemotherapy patients’ tissue can be grouped as damaged tumour cells (completely necrotic or degenerative, vascular lesions (engorged, cystic, damaged blood vessels, associated with haemorrhages) and reparative changes involving fibroblast, angioblast, and osteoblast. In the case of complete destruction of tumour cells, the cells are absent or barely visible and sometimes recognised as bizarre cells. Although pre-operative chemotherapy samples show different changes from non-chemotherapy samples, no complete discrimination between those two can be reached. Probably the changes observed in pre-operative chemotherapy samples are a sum of spontaneous and chemotherapy-induced necrosis; we categorized all as the result of the chemotherapy [20].

**Fig. 2:**
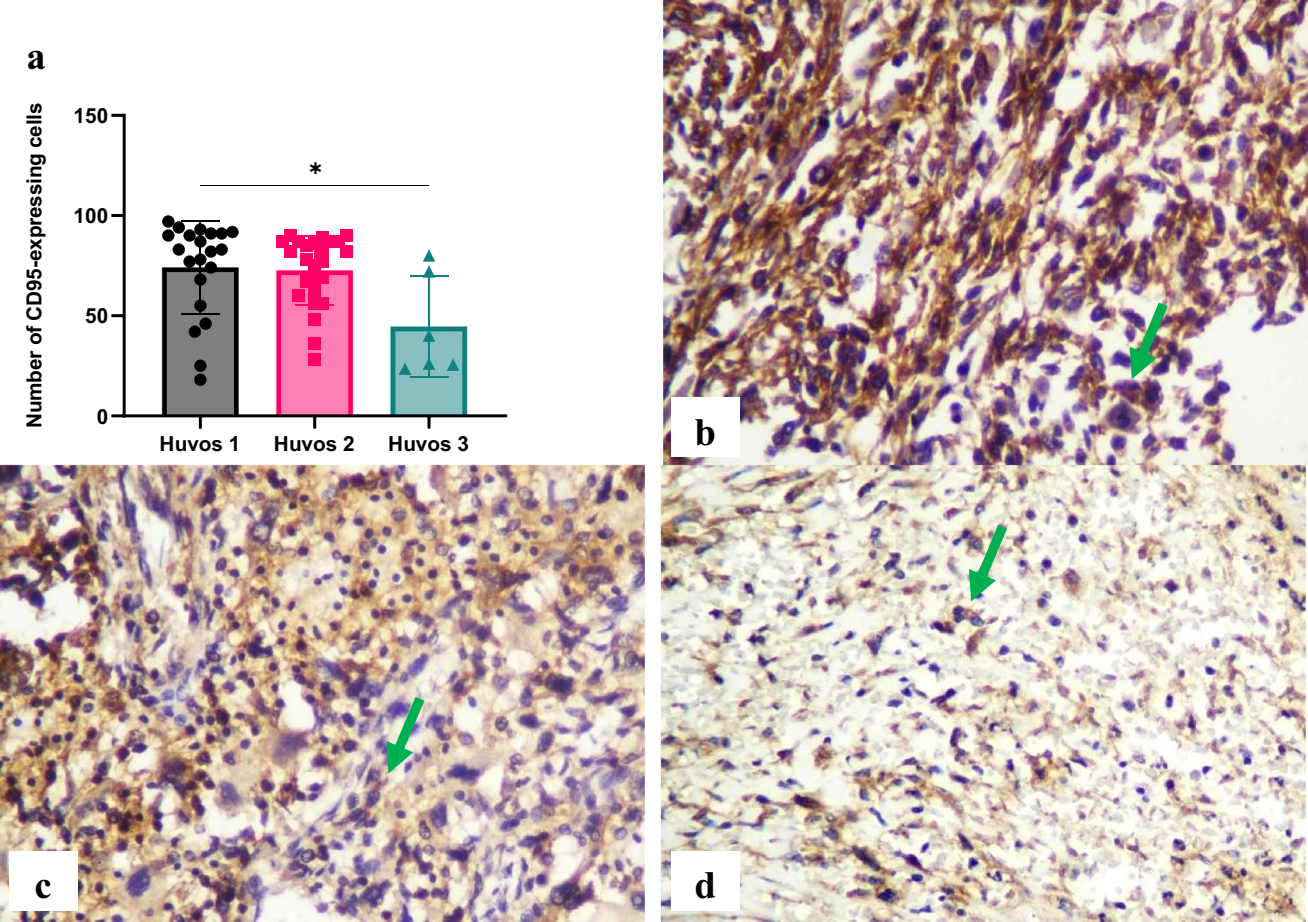
Immunohistochemistry of CD95 receptor (CD95R) in osteosarcoma cell. The number of CD95R-expressing cells was evaluated using immunohistochemistry specimens and a light microscope at 400 × magnification. Significant differences were observed between Huvos grade I and Huvos grade III groups (*p < 0.05) (a). The representative immunohistochemistry results illustrate Huvos grade I (b), Huvos grade II (c), and Huvos grade III groups (d). Green arrows indicate positive staining cells

A significant difference (*p* < 0.05) in CD95R expression in lymphocytes was identified between poor and good chemotherapy responses in osteosarcoma. CD95R expressions were higher in the poor response group (74.09 ± 23.30) compared to the moderate (72.60 ± 17.32) and good (44.53 ± 25.19) response groups, and the moderate response group exhibited higher levels than the good response group (Fig. 2).

**Fig. 3:**
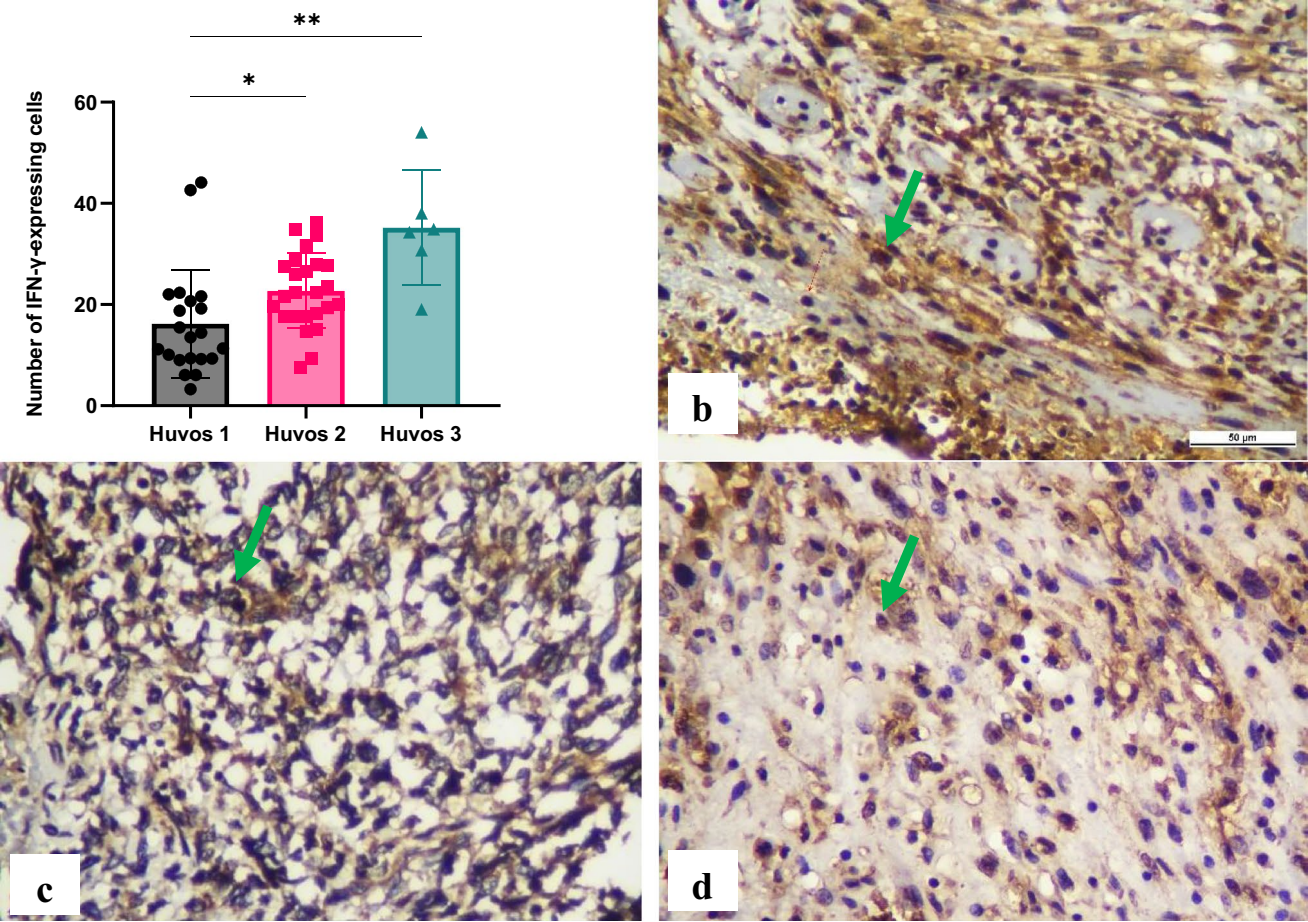
Immunohistochemical expression of interferon (IFN)-γ in lymphocytes. The number of IFN-γ-expressing cells was counted using immunohistochemistry specimens and a light microscope at 400 × magnification. Significant differences were observed between groups (*p < 0.05, **p < 0.01) (a). The representative immunohistochemistry results illustrate Huvos grade I (b), Huvos grade II (c), and Huvos grade III groups (d). Green arrows indicate positive staining cells

Significant differences (*p* < 0.05) in IFN-γ expression in lymphocytes were observed among the chemotherapy response groups in osteosarcoma, including between poor and moderate responses, as well as poor and good responses. Higher IFN-γ expressions were noted in the good response group (35.15 ± 11.36) compared to the moderate (22.69 ± 7.439) and poor (16.17 ± 10.66) response groups, and the moderate response group exhibited higher levels than the poor response group (Fig. 3).

**Fig. 4:**
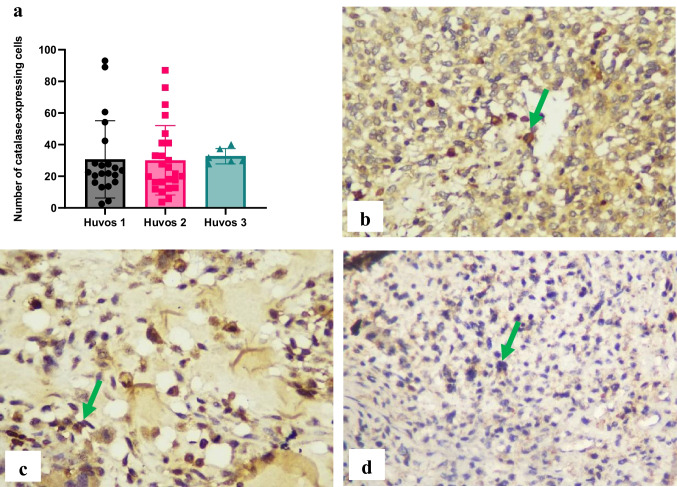
Immunohistochemical expression of catalase in osteosarcoma cells. The number of catalase-expressing cells was evaluated using immunohistochemistry specimens and a light microscope at 400 × magnification. There were no significant differences between groups (a). The representative immunohistochemistry results illustrate Huvos grade I (b), Huvos grade II (c), and Huvos grade III groups (d). Green arrows indicate positive staining cells

No significant differences (*p* > 0.05) in catalase expression in osteosarcoma cells were observed among the chemotherapy response groups in osteosarcoma. Catalase expressions were 30.68 ± 24.40 in the poor response group, 30.06 ± 21.95 in the moderate response group, and 32.70 ± 4.856 in the good response group (Fig. 4).

**Fig. 5:**
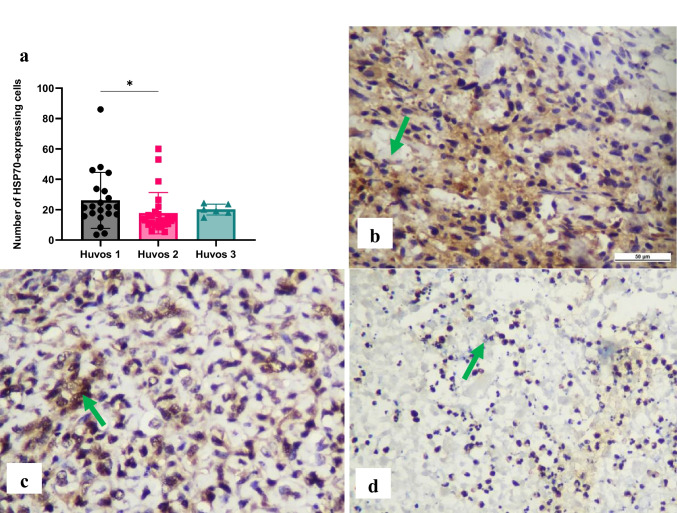
Immunohistochemical expression of heat-shock protein (Hsp)70 in osteosarcoma cells The number of Hsp70-expressing cells was evaluated using immunohistochemistry specimens and a light microscope at 400 × magnification. There was a significant difference between Huvos grade I and Huvos grade II groups (*p < 0.05) (a). The representative immunohistochemistry results illustrate Huvos grade I (b), Huvos grade II (c), and Huvos grade III groups (d). Green arrows indicate positive staining cells

A significant difference (*p* < 0.05) was observed in Hsp70 expression in osteosarcoma cells between poor and moderate chemotherapy responses in osteosarcoma. The highest Hsp70 expression was observed in the poor response group (26.11 ± 18.40) compared to the moderate (17.73 ± 13.58) and good (20.12 ± 3.549) response groups (Fig. 5).

**Fig. 6:**
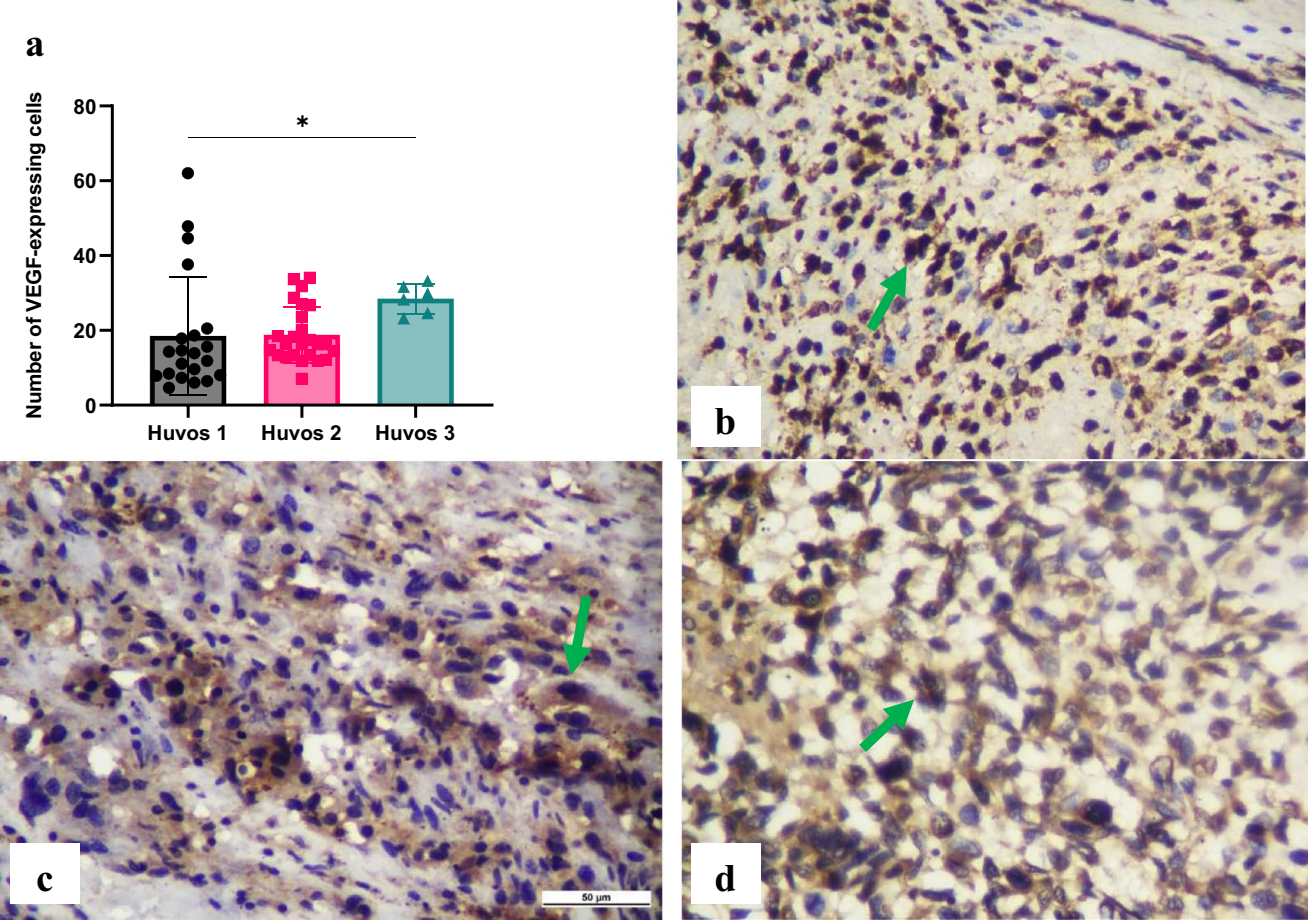
Immunohistochemical expression of vascular endothelial growth factor (VEGF) in macrophages. The number of VEGF-expressing cells was evaluated using immunohistochemistry specimens and a light microscope at 400 × magnification. There was a significant difference between Huvos grade I and Huvos grade II groups (*p < 0.05) (a). The representative immunohistochemistry results illustrate Huvos grade I (b), Huvos grade II (c), and Huvos grade III groups (d). Green arrows indicate positive staining cells

A significant difference (*p* < 0.05) in VEGF expression in macrophages was found between the poor and good chemotherapy responses in osteosarcoma, with higher VEGF expression in the good response group. VEGF expressions were 18.49 ± 15.80 in the poor response group, 18.77 ± 7.481 in the moderate response group, and 28.45 ± 3.917 in the good response group (Fig. 6).

## Discussion

The incidence of osteosarcoma in this study was highest in the age range of 10–19 years and lowest above 30 years. This pattern aligns with another study that noted most osteosarcoma cases typically occur in children aged 12–16 years [19] and aligns with the notion that most osteosarcoma cases develop between the ages of 14–18 years old, corresponding to the acceleration of pubertal growth. The annual incidence rate is 4.4 cases/1 million population for people aged 0–24 years, with males affected more frequently and the M:F ratio 1.3: 1 [21].

Osteosarcoma is highly malignant and shows strong invasive capabilities, progressive disease development, and a very high mortality rate [22]. Before the discovery of chemotherapy in the 1970s, the prognosis for osteosarcoma patients was deemed very poor, with a survival rate of < 20%. The management involving surgical resection with adequate surgical margins combined with neoadjuvant chemotherapy has increased the survival rate to 60–70% and has reached a plateau. The survival rate for patients with localized osteosarcoma is 65%, while for patients with metastatic osteosarcoma, it is < 20% [23, 24]. In selective cases, patients can be rescued by metastasectomy [25].

Recent approaches using multimodal strategies, including systemic chemotherapy before surgery (neoadjuvant chemotherapy), followed by local surgical procedures and supplemented with postoperative chemotherapy (adjuvant chemotherapy), have improved long-term success rates to 60–70%, enhancing overall survival [26]. However, current therapeutic modalities have various disadvantages, such as the lack of a significant improvement in survival rates despite decades of chemotherapy use, and the side effects associated with high-dose chemotherapy. Given the advancements in scientific knowledge, there is a fundamental need to identify new biological indicators for patient prognosis and chemotherapy response detection. The aim is also to develop innovative and specific therapeutic approaches targeting specific molecular targets (targeted therapy) to improve outcomes for osteosarcoma patients with a poor prognosis [27].

Only four chemotherapy agents have shown objective responses in osteosarcoma: Doxorubicin at 43%, Ifosfamide at 33%, Methotrexate at 32%, and Cisplatin at 26%. Studies utilizing three active agents have demonstrated better outcomes than those using only two regimens [28]. The first-line chemotherapy includes Cisplatin and Doxorubicin (category 1), MAP (high-dose Methotrexate, Cisplatin, and Doxorubicin), and the combination of Doxorubicin, Cisplatin, Ifosfamide, and high-dose Methotrexate (MAPI) [5]. In this study, patients received neoadjuvant chemotherapy following the National Comprehensive Cancer Network (NCCN) guidelines, consisting of Cisplatin and Doxorubicin only. The mean 5-year event-free survival (EFS) and 5-year overall survival (OAS) are 48% and 62% for two-drug regimens, and 58% and 70% respectively for three or more drug regimens. Patients treated with chemotherapy have lower 5y-EFS compared to those who are treated with surgery and chemotherapy, showing that patients with localised high-grade osteosarcoma cannot be treated by chemotherapy alone [29]. Intraarterial cisplatin is more effective than methotrexate. Multidrug regimen without methotrexate achieves similar survival rates to methotrexate-based regimens. The meta-analysis on EFS shows significantly improved survival by administering three drug-regimen compared to a two-drug regimen but shows no significant difference with a 4 drug-regimen [29].

The long-term success of traditional chemotherapy and targeted anticancer agents often relies on immunological effects. Immunogenicity results from a combination of antigenicity and adjuvanticity, and many anticancer drugs activate adaptive stress responses in malignant cells, serving as immunological adjuvants [30]. In some malignancies, both innate and adaptive immune cells play a role in the tumour microenvironment, involving natural killer (NK) cells, antigen-presenting cells (APC) like macrophages and dendritic cells (DC), and lymphocytes, effectively controlling tumours [31].

Cell death plays a pivotal role in cancer, encompassing both programmed cell death and non-programmed cell death. Programmed cell death involves apoptosis, autophagy, and necroptosis (programmed necrosis) [32]. The apoptosis process unfolds through two pathways: extrinsic (receptor-mediated apoptosis) and intrinsic (mitochondria-mediated apoptosis) [33]. The extrinsic pathway is initiated by the binding of death receptors, such as CD95R (and analogous receptors like tumour necrosis factor (TNF) receptor 1 and its counterparts), on the plasma membrane with its extracellular ligand, CD95L [32]. Upon stimulation, CD95L binds to CD95R, forming a death signal complex. Following its formation, the death-inducing signalling complex (DISC) initiates, recruiting various proapoptotic factors, including caspase-8. Subsequent cleavage of caspase-8 and effector caspase-3 leads to proteolytic protein breakdown and apoptosis [34–36].

In this study, a significant difference was found between poor and good responses, with the highest CD95R expression in poor responses, suggesting that higher CD95R expression corresponds to increased chemotherapy resistance. This contradicts previous studies indicating that low CD95R expression is associated with a worse prognosis in osteosarcoma patients [37]. CD95R expression is inversely related to tumour metastasis potential, with increased CD95R expression correlated with decreased tumour cell metastasis potential [38]. This is supported by studies showing prolonged survival trends in patients with CD95R expression [39].

CD95R, expressed on the tumour cell surface, contributes to immune cell-mediated cytotoxicity. Patients with a poor chemotherapy response have low CD95R levels, making it easier for tumour cells to metastasise. Those with high CD95R levels have the potential for longer survival and a lower probability of death [39]. Another study investigated the effects of Doxorubicin and Edelfosine lipid nanoparticles on osteosarcoma. The study revealed that this chemotherapy combination increased CD95R expression on tumour cell surfaces, triggering apoptosis [40].

CD95R is associated with the TNF receptor family and mediates apoptosis when bound to its natural ligand, CD95L (CD178/TNFSF6), or stimulated with agonistic antibodies [35]. CD95L is predominantly expressed in activated T lymphocytes and NK cells, the primary subset of innate immune cells responsible for antiviral and antitumor responses [41, 42]. NK cells, defined as CD56^+^/CD3.^−^ lymphocytes, exhibit cytotoxicity, and cytokine production, particularly in response to exogenous cytokines like IL-2, IL-12, IL-15, IL-18, and IL-21 [43–46]. Among the most prominent cytokines produced by NK cells are TNF-α and IFN-γ [47].

IFN-γ, a crucial cytokine, enhances tumour cell death mediated by CD95R, playing a vital role in immunomodulation and antitumor activity [48]. IFN-γ is an immunomodulatory cytokine that promotes apoptosis directly or through induced lipid peroxidation and ferroptosis, contributing to an effective antitumor immune response [49]. This is supported by a study that evaluated the influence of IFN-γ on ligand expression in various NK cells on cancer cells, with none of these ligands being downregulated by IFN-γ. However, CD274/PD-L1, CD54/ICAM-1, HLA-DR, MHC class I, CD95/FasR, and CD270/HVEM are regulated in various tumour types [34]. More specifically, in osteosarcoma, a study mentioned that IFN-γ sensitizes osteosarcoma cells to CD95R, inducing apoptosis through the upregulation of CD95R and caspase-8 [50, 51].

This study revealed significant differences in IFN-γ expression among chemotherapy response groups. This suggests that IFN-γ might play a key role in the effectiveness of chemotherapy, with lower levels associated with increased resistance. The results are in line with a previous study that suggested a correlation between elevated PSMD14 expression and a higher-risk category (younger age group, ≤ 20.83 years of age), metastasis within five years, and a higher tumour grade in osteosarcoma patients [37]. PSMD14 is a component of the 26S proteasome. The group with increased PSMD14 expression exhibited decreased immune responses, including reduced levels of IFN-γ [52].

A previous study emphasized the significant contribution of IFN-γ to antitumor and antimetastatic effects, triggering FAS ligand formation and apoptosis induction in cancer cells [50]. Interestingly, previous studies highlighted the positive impact of chemotherapy on IFN-γ levels, demonstrating its correlation with good treatment response in osteosarcoma [53]. Additionally, a previous study underscores the potential preventive role of NK cells in osteosarcoma development, as evidenced by higher circulating NK cell counts in normal controls compared to osteosarcoma patients [54, 55]. Natural Killer (NK) cells release IFN-γ as one of the most potent effector cytokines [47].

Our study suggests that in a good response, the immune system remains resilient against chemotherapy, marked by an increase in IFN-γ. The phenomenon of decreasing CD95R expression from poor to good responses, accompanied by an increase in IFN-γ, suggests a declining number of viable osteosarcoma cells as the Huvos grading advances.

Excessive reactive oxygen species (ROS) leading to oxidative stress plays a pivotal role in carcinogenesis [56]. Cells maintain intracellular homeostasis by developing an extensive antioxidant system, including catalase, superoxide dismutase (SOD), and glutathione peroxidase (GPx). Catalase facilitates the decomposition of hydrogen peroxide into water and oxygen (2H_2_O_2_ → 2H_2_O + O_2_), crucial for degrading H_2_O_2_ and preserving cells against oxidative damage. The expression of catalase varies in tumour cells [57]. Low catalase expression correlates with high H_2_O_2_ production, influencing signalling pathway activation to induce proliferation, migration, and invasion in cancer cells [58–60]. Changes in catalase expression after short-term H_2_O_2_ exposure are influenced by factors such as exposure time, H_2_O_2_ concentration, basal antioxidant enzyme capacity, and the cellular model used [57]. High catalase expression has been observed in certain human cancer cell lines, including gastric cancer exposed to cisplatin chemotherapy [61].

No significant difference in catalase expression was observed among the chemotherapy response groups, implying that catalase may not play a decisive role in chemotherapy outcomes. Similarly, a previous study conducted on male patients under 20 years old with osteosarcoma did not find a significant association between catalase level and chemotherapy response [62]. However, its consistently elevated expression in poor responders raises the need for further investigation. Catalase expression did not exhibit the capacity to protect against ROS attacks induced by chemotherapy.

The role of Hsp in maintaining cellular homeostasis and protection is vital, with their heightened production occurring under stressful conditions [63]. These proteins play a crucial role in safeguarding cells from stress-related injuries and are often overexpressed in various malignancies [64]. Among them, Hsp70, an ATP-dependent molecular chaperone, regulates protein conformation, stability, and interactions, including essential proteins for cell survival, growth, and immune responses [65, 66]. Excessive Hsp70 expression in cancer cells is implicated in various oncogenic events, such as anti-apoptotic responses, antitumor immune responses, tumour growth, and cell migration [67].

The poor response group exhibited the highest Hsp70 expression. Previous studies on Hsp in osteosarcoma revealed prognostic associations, with Hsp27, Hsp60, and Hsp70 linked to poor prognosis [68]. Additionally, Hsp27 and Hsp70 were identified as potential markers to distinguish conventional and low-grade central osteosarcoma [64]. Lower Hsp70 expression correlated with higher tumour cell necrosis rate (TCNR), indicating its utility in predicting and evaluating the neoadjuvant chemotherapy's effectiveness in inhibiting osteosarcoma cell proliferation [69]. These findings contribute valuable insights into the complex interplay of Hsp70 in osteosarcoma and its response to chemotherapy, providing avenues for further study and potential therapeutic interventions.

Angiogenesis is a crucial aspect of tumorigenesis, influencing tumour growth and metastatic potential [70]. Vascular endothelial growth factor (VEGF), a key player in angiogenesis and vasculogenesis, primarily acts on various cell types, particularly endothelial cells [71]. VEGF is considered a major mediator of angiogenesis and has been implicated in carcinogenesis and metastasis [72]. Tumour malignancy requires a persistent supply of new blood vessels to sustain growth and facilitate metastasis [73]. In osteosarcoma, VEGF-A expression, as demonstrated by immunohistochemistry, has been associated with a higher risk of lung metastasis and poorer survival [74].

In our study, VEGF expression exhibited distinct patterns, notably higher in good response cases compared to both moderate and poor responses. This variability may be attributed to significant cell death in osteosarcoma, hindering the formation of new blood vessels. Despite numerous studies exploring the correlation between VEGF overexpression and clinical outcomes in osteosarcoma patients, the results remain inconclusive. Meta-analyses assessing the connection between VEGF expression and overall survival in osteosarcoma patients have consistently indicated that elevated VEGF expression is associated with poorer overall survival, with no significant heterogeneity among studies [72]. The positive expression of VEGF in surviving tumour cells following neoadjuvant chemotherapy emerges as a prognostic factor in osteosarcoma, offering valuable insights for identifying potential angiogenic targets in the pursuit of personalized future therapies [75].

We studied the expression on resected post-chemo specimens. Osteosarcoma is an extremely heterogeneous tumour and diagnostic (core-needle) biopsies are only small specimens, which are taken for diagnosis and do not necessarily reflect the expression of molecules throughout the whole tumour. Our study focuses on the response to chemotherapy correlated with immunological response. Including paired biopsy specimens would not result in reliable data given the tumour heterogeneity, so the results reflect the expression profile of the surviving cells post-chemotherapy. Pre-chemo expression in the tumours and changes over time during chemotherapy treatment are not assessed in this study.

The novelty of this study lies in the conventional chemotherapy's mode of action, typically involving DNA synthesis inhibition through folate metabolism disruption (methyl tetrahydrofolate). However, it appears that chemotherapy can also influence the immune response, as evidenced by the findings of increased IFN-γ expression and decreased CD95R expression in a good response to chemotherapy.

Considering our findings, suggestions for further study and clinical implications emerge. There is a need for further investigations to delve into the specific functions of lymphocytes in the pathogenesis of osteosarcoma malignancy. Understanding the intricate interplay between the immune system and osteosarcoma progression can pave the way for targeted therapeutic interventions and personalized treatment strategies. Expanding our knowledge in these areas will undoubtedly contribute to refining prognostic assessments and advancing the development of more effective therapeutic approaches for osteosarcoma patients.

## Conclusion

Our study on osteosarcoma chemotherapy responses and the associated molecular expressions provides valuable insights into mechanisms of resistance and potential routes for exploration for additional treatment options. The findings contribute to the growing understanding of the complex interactions between the immune system, antioxidants, and chemotherapy responses in osteosarcoma patients. The identification of specific biomarkers associated with treatment response provides a foundation for potential targeted therapeutic interventions in the pursuit of improved patient outcomes.

## Data Availability

The datasets generated during and/or analysed during the current study are available from the corresponding author upon reasonable request.
